# Die bundesweiten Maßnahmen zur Alkoholprävention der Bundeszentrale für gesundheitliche Aufklärung (BZgA)

**DOI:** 10.1007/s00103-021-03333-w

**Published:** 2021-05-31

**Authors:** Tobias Schwarz, Michaela Goecke

**Affiliations:** Referat 1-13, Prävention des Substanzmissbrauchs, Suchtprävention, Bundeszentrale für gesundheitliche Aufklärung (BZgA), Maarweg 149–161, 50825 Köln, Deutschland

**Keywords:** Gesundheitskompetenz, Mehrebenenkampagne, Personalkommunikation, Digitale Kommunikation, Massenkommunikation, Health competence, Multilevel campaign, Personal communication, Digital communication, Mass communication

## Abstract

Im europäischen Vergleich ist der Alkoholkonsum in Deutschland nach wie vor hoch. Eine langfristige Senkung kann zum Rückgang alkoholbedingter Morbidität und Mortalität beitragen. Die Bundeszentrale für gesundheitliche Aufklärung (BZgA) fokussiert seit vielen Jahren innerhalb der Suchtprävention die Alkoholprävention und setzt dazu 3 zielgruppenspezifische bundesweite Mehrebenenkampagnen um. Die Kampagne „Null Alkohol – Voll Power“ richtet sich an Jugendliche im Alter von 12 bis 16 Jahren, die Kampagne „Alkohol? Kenn dein Limit.“ besteht aus 2 Teilkampagnen und richtet sich an 16- bis 20-Jährige sowie an Erwachsene. Außerdem bietet die BZgA Sportvereinen die Möglichkeit zur Teilnahme an der Aktion „Alkoholfrei Sport genießen“. In diesem Beitrag werden die Maßnahmen und die dahinterstehenden Konzepte vorgestellt.

Die Kampagnen sind miteinander im Sinne einer Präventionskette verbunden, sodass Synergien genutzt werden können. Sie basieren auf den Kriterien des Social-Marketings und unterliegen einem kontinuierlichen Qualitätssicherungsprozess. Um die Zielgruppen zu erreichen, wird primär die Internetkommunikation genutzt, die ergänzt wird durch personalkommunikative Angebote in Lebenswelten sowie Massenkommunikation in Form von Plakaten, Spots, Anzeigen und Printmedien. Bei der Umsetzung von Angeboten in Schulen, Vereinen oder Kommunen setzt die BZgA auf eine gute Kooperation und Abstimmung mit den Ländern. Als begleitendes Monitoring führt sie seit vielen Jahrzehnten regelmäßig Repräsentativbefragungen u. a. zum Alkoholkonsumverhalten der 12- bis 25-Jährigen in Deutschland durch. Die Ergebnisse zeigen, dass der Alkoholkonsum im langfristigen Trend in Deutschland zwar rückläufig, aber dennoch hoch ist.

## Abstract (en)

Alcohol consumption in Germany continues to be high relative to its European neighbors. A long-term reduction of alcohol consumption can result in a reduction of the morbidity and mortality associated with alcohol. For many years now, the Federal Centre for Health Education (BZgA) has directed its focus in the field of addiction prevention on alcohol prevention and implemented three national multilevel campaigns intended for this target group. “Null Alkohol – Voll Power” (No Alcohol – Full Power) addresses adolescents between the ages of 12 and 16 and the campaign “Alkohol? Kenn dein Limit.” (Alcohol? Know Your Limit.) comprises two separate campaigns dedicated to adults and 16- to 20-year-olds, respectively. Further, the BZgA provides sports clubs with the opportunity to participate in a program entitled “Alkoholfrei Sport genießen” (Enjoying Sports Without Alcohol). In this contribution, these measures and their foundational concepts are presented.

The campaigns are interconnected to form a prevention chain that serves to bundle the individual efforts. All are based on the principles of social marketing and are continually quality checked. At the center of these efforts lies internet communication, complemented by direct communication in the lives of the target groups as well as mass communication in the form of billboards, TV and radio spots, ads, and print media. The BZgA is able to implement these programs in schools, clubs, and at the municipal level because of the good cooperation and coordination it maintains with the individual federal states. For decades now, the BZgA has conducted monitoring in the form of regular representative surveys, among others, on the subject of alcohol consumption behavior among 12- to 25-year-olds in Germany. The results show that although overall alcohol consumption may be on the decline over the long term, it still remains high.

## Einleitung

Alkoholkonsum ist einer der weltweit führenden Risikofaktoren für Morbidität und Mortalität [1, 2]. Langfristig ging der Alkoholkonsum in Deutschland in den vergangenen Jahren zwar kontinuierlich zurück: 2019 konsumierten 32,9 % der 18- bis 25-Jährigen mindestens wöchentlich Alkohol, 2004 waren es noch 43,6 % [3] (zu weiteren Prävalenzen s. Beiträge von Orth und Merkel sowie Kraus et al. in diesem Themenheft). Aber mit einem jährlichen Pro-Kopf-Konsum (der Bevölkerung ab dem Alter von 15 Jahren) von Reinalkohol in Höhe von 10,9 l liegt Deutschland immer noch deutlich über dem Durchschnitt der Mitgliedstaaten der Organisation für wirtschaftliche Zusammenarbeit und Entwicklung (OECD) von 8,9 l [4].

Aufgrund des Zusammenhangs zwischen der Konsummenge und dem Ausmaß alkoholbedingter Erkrankungen kann die langfristige bevölkerungsweite Senkung des Pro-Kopf-Konsums eine wirkungsvolle Strategie sein, um einen Rückgang der alkoholbezogenen Schäden, d. h. körperliche und psychische Erkrankungen, Unfälle, Arbeitsausfälle usw., zu erreichen, wie dies z. B. auch im Nationalen Gesundheitsziel „Alkohol reduzieren“ 2015 formuliert wurde [5]. Als besonders wirkungsvoll zur Verringerung des Alkoholkonsums und damit zur Prävention von alkoholbedingter Morbidität und Mortalität gilt die Kombination von Maßnahmen der Verhaltens- und Verhältnisprävention, also etwa die Beschränkung der Verfügbarkeit oder die Erhöhung der Preise für alkoholische Getränke in Kombination mit Aufklärung über die Gesundheitsrisiken durch deren Konsum.

**Figure 1:**
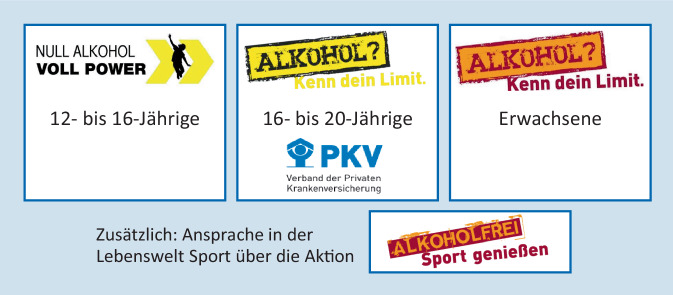

Die vorrangige Aufgabe der Bundeszentrale für gesundheitliche Aufklärung (BZgA) ist dabei die dauerhafte Aufklärungs- und Informationsarbeit. Seit dem Jahr 2009 liegt daher ein Schwerpunkt auf der Prävention des Alkoholmissbrauchs (im Folgenden „Alkoholprävention“). Aktuell werden 3 Kampagnen zur Alkoholprävention durchgeführt („Null Alkohol – Voll Power“ für 12- bis 16-Jährige, „Alkohol? Kenn dein Limit.“ für 16- bis 20-Jährige bzw. die gleichnamige Kampagne für Erwachsene) sowie das Programm „Alkoholfrei Sport Genießen“ in Kooperation mit Breitensportverbänden (Abb. 1). Ab dem Alter von 12 Jahren werden alle Altersjahrgänge sowie beide Geschlechter gleichermaßen adressiert, wobei ein deutlicher Schwerpunkt auf den Jugendlichen und jungen Erwachsenen im Alter zwischen 16 und 20 Jahren mit der größten bundesweiten Alkoholpräventionskampagne „Alkohol? Kenn dein Limit.“ liegt. Innerhalb der erwachsenen Allgemeinbevölkerung ab 21 Jahren liegen die Schwerpunkte auf Eltern, Schwangeren und ihrem Umfeld, suchtbelasteten Familien sowie auf älteren Menschen. Damit wird eine Präventionskette geschaffen, die alle Altersgruppen und bestimmte Teilpopulationen differenziert in den Blick nimmt, eine entsprechend abgestufte Zielsetzung aufweist und deren Elemente sich hinsichtlich Kommunikation und Themenwahl entsprechend unterscheiden und ergänzen. Wichtige Multiplikatoren/innen, die in die Kampagnen einbezogen werden, sind u. a. Eltern, Lehrkräfte sowie Ärzte/innen.

Ziel dieses Beitrags ist es, die wesentlichen Bestandteile der BZgA-Alkoholprävention überblicksweise vollständig darzustellen, um zu zeigen, dass grundsätzlich unterschiedliche Interventionsformen auf verschiedenen Ebenen innerhalb eines Gesamtkonzepts zusammenwirken. Es soll deutlich werden, dass dabei adressatengerecht ausdifferenzierte Maßnahmen aufeinander aufbauen und sich ergänzen. Nach der Darstellung des für alle 3 Kampagnen einheitlichen konzeptionellen Rahmens sowie der diese flankierenden Aktivitäten werden die Kampagnen mit ihren jeweiligen Zielen und Adressaten/innengruppen genauer beschrieben. Auf die konkreten Maßnahmen der Internetkommunikation, der Personalkommunikation sowie der Printmedien und Massenkommunikation gehen die dann folgenden 3 Kapitel ein. Dort wird gezeigt, wie sich die Kampagnenangebote jeweils adressatenspezifisch unterscheiden bzw. ergänzen.

## Das Kampagnenkonzept der BZgA

**Figure 2:**
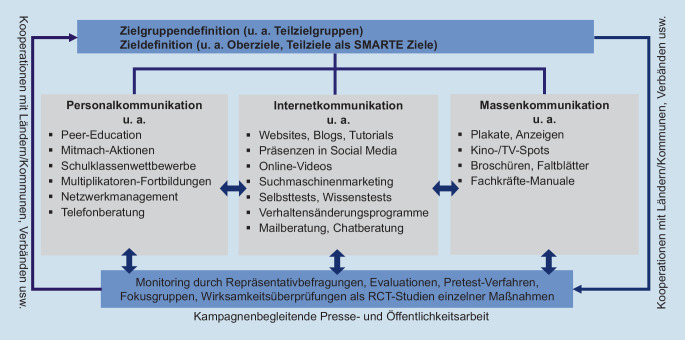

Der Aufbau der 3 BZgA-Kampagnen zur Alkoholprävention folgt dem Konzept der Mehrebenenkampagne der BZgA [6, 7]. Unter *Kampagne* wird dabei ein Maßnahmenbündel verstanden, das über mehrere Jahre kontinuierlich durchgeführt wird. Da in Deutschland als Hochkonsumland nicht mit kurzfristigen Verhaltensänderungen in der Bevölkerung zu rechnen ist, kann die Konsumreduktion nur in einem mittel- bis langfristigen Prozess erfolgen. Dieser sollte, dem Vorschlag der Weltgesundheitsorganisation (WHO) folgend, mit Aufklärungs- und Informationskampagnen dauerhaft unterstützt werden [8]. Von mehreren *Ebenen* wird gesprochen, weil die 3 Interventionsbereiche digitale Kommunikation, Personalkommunikation, Printmedien/Massenkommunikation systematisch miteinander kombiniert werden (Abb. 2). Dem liegt eine Einsicht aus dem Social Marketing, also dem Einsatz von Maßnahmen des klassischen Marketings, mit u. a. gesundheitspolitischen Zielen [9] zugrunde, wonach personalkommunikative Maßnahmen, z. B. Interventionen in Schulen o. Ä., von den Endadressaten/innen besser angenommen werden, wenn der Absender bereits bekannt und akzeptiert ist. Gleichzeitig stützen persönliche, individuelle Gespräche mit einer realen Person sowie der bidirektionale Kontakt über die sozialen Medien die massenmedialen Interventionen [10].

Neben dem analogen Aufbau basieren die 3 Alkoholpräventionskampagnen auch auf einem einheitlichen theoretischen Konzept. Die Kampagnen der BZgA sind systematisch entsprechend dem Public Health Action Cycle (gesundheitspolitischer Aktionszyklus) geplant [11]. Grundlegend für die Kampagnenkonzepte ist der Bezug auf sozialkognitive Modelle des Gesundheitsverhaltens, die verschiedene handlungsleitende Faktoren zueinander in Beziehung setzen (individuelle Einstellungen, das Wissen über mögliche Gesundheitsrisiken und das individuell wahrgenommene Risiko, die Verhaltensabsichten, den gesellschaftlichen Kontext usw.). Beispiele für solche Modelle sind die „Theorie des Geplanten Verhaltens“ [12] und das „Sozialkognitive Prozessmodell des Gesundheitsverhaltens“ (HAPA [Health Action Process Approach] [13]). Basierend auf diesen Modellen sollen die Kampagnen u. a. das individuell verfügbare Gesundheitswissen erhöhen, denn dieses bildet die Grundlage informierter Gesundheitsentscheidungen. Zudem sollen Selbstreflexions- und Kommunikationsprozesse unter den (potenziellen) Konsumierenden ausgelöst werden, um zur Normkorrektur beizutragen. Dazu transportieren die Kampagnen positive Botschaften, die den Gewinn durch Verhaltensänderungen hervorheben (Gain-Framing) und appellieren an das Verantwortungsbewusstsein der Endadressaten/innen im Hinblick auf ihren Alkoholkonsum, was durch Erhöhung der Motivation unterstützt werden kann [14].

**Figure 3:**
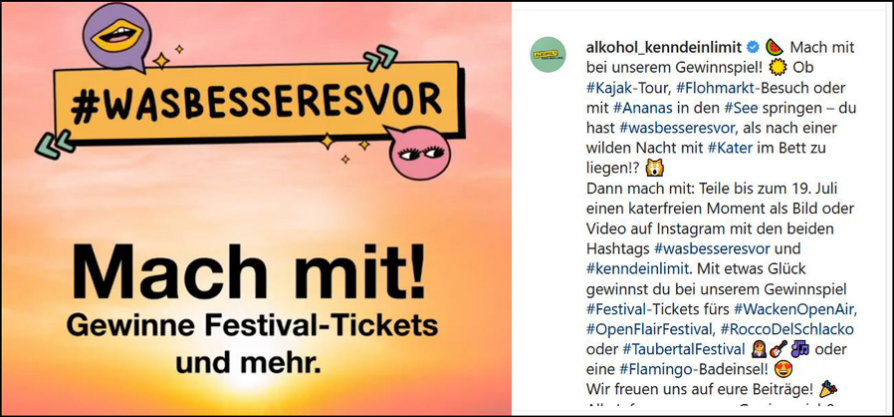

Die Kampagnen ergänzen sich je nach Adressatenkreis inhaltlich und verweisen, wenn sinnvoll, aufeinander – so werden beispielsweise in der Jugendkampagne vor allem die kurzfristigen Risiken des Alkoholkonsums wie etwa die Unfallgefahr und der Ansehensverlust in der Peergroup thematisiert, in der Erwachsenenkampagne die potenziellen langfristigen Gesundheitsschäden, wie etwa das erhöhte Krebsrisiko. Zur aktivierenden Ansprache der Endadressaten/innen werden zu diesen Inhalten Gain-Frames entwickelt, wie etwa die Mitmachaktion „Was besseres vor“ der Jugendkampagne (Abb. 3).

Die Kampagneninhalte werden zudem laufend mit Angeboten in den Bundesländern abgestimmt bzw. werden spezifische Bedarfe, etwa auf kommunaler Ebene, u. a. über die bundesweite Vernetzung der BZgA mit den Länderkoordinationen zur Suchtprävention ermittelt. Daraus entstehende Neuentwicklungen mit Modellcharakter, wie zum Beispiel der „KlarSicht-Koffer“, die flexibel einsetzbare Koffervariante des „KlarSicht“-Mitmachparcours (s. unten), werden nach einer Testphase in die eigenständige Durchführung durch die Bundesländer transferiert.

Die Erreichung der Endadressaten/innen wird neben der direkten Ansprache (Internet- und Personalkommunikation) durch den systematischen Einbezug von Fachkräften (etwa Präventionsfachkräften, Lehrkräften, Sporttrainer/innen) bzw. durch Kooperationen mit entsprechenden Verbänden (etwa der Schwangerenvorsorge und Sportverbänden) unterstützt, die kampagnenübergreifend aufeinander abgestimmt sind. Mittels Handreichungen und Weiterbildungsangeboten für Fachkräfte (zum Beispiel in Schulen oder in der kommunalen Suchtprävention) fördert die BZgA die Qualität dezentraler Alkoholpräventionsaktivitäten. Die Kampagnen zur Alkoholprävention werden darüber hinaus durch Maßnahmen der BZgA zur frühen Resilienzförderung im Kindesalter („Kinder stark machen“) ergänzt. Nicht zuletzt werden alle Elemente der BZgA-Alkoholprävention außerdem durch eine gemeinsame Presse- und Öffentlichkeitsarbeit begleitet.

Erkenntnisse aus den periodischen Monitorings (BZgA-Studien „Drogenaffinitätsstudie“, die seit 1973 alle 3–4 Jahre durchgeführt wird, sowie der seit 2010 alle 2 Jahre durchgeführte „Alkoholsurvey“ [3, 15]) werden von den Alkoholpräventionskampagnen gemeinsam genutzt. Zur Qualitätssicherung werden für alle Einzelmaßnahmen Struktur‑, Prozess- oder Ergebnisevaluationen durchgeführt [16–24], die zur Weiterentwicklung etwa des Facebook-Kanals der Jugendkampagne oder des „KlarSicht“-Koffers genutzt wurden. Durch Ergebnisevaluationen konnten empirische Wirksamkeitsnachweise für Einzelmaßnahmen erbracht werden, die im Rahmen der Kampagne neu entwickelt wurden. Durch eine experimentelle Kontrollgruppenstudie zum Einfluss der ersten Plakatmotive der Jugendkampagne auf Alkoholwirkungserwartungen wurde ermittelt, dass die impliziten Einstellungen gegenüber Alkoholwirkungen durch die Wahrnehmung der Motive negativer wurden [18]. Eine clusterrandomisierte Studie über den BZgA-Ansatz, junge Erwachsene als Peer-Edukatoren zur Alkoholprävention einzusetzen, ergab, dass Jugendliche nach einem Gespräch mit den Kampagnen-Peers besser über die Risiken des Alkoholkonsums informiert waren und sich darin bestärkt fühlten, alkoholische Getränke abzulehnen [21]. Eine weitere clusterrandomisierte Kontrollgruppenstudie zum bundesweiten Klassenwettbewerb „Klar bleiben“ ergab, dass die Jugendlichen mit vorheriger Konsumerfahrung nach der Teilnahme seltener und weniger Alkohol konsumierten [23].

Die Motive der Plakat- und Anzeigenschaltungen werden durch Pretests auf Verständlichkeit und Akzeptanz von Motiven und Botschaften geprüft. Dadurch sowie durch sich verändernde Rahmenbedingungen und strategische Schwerpunktsetzungen befinden sich die Kampagnen in einem kontinuierlichen Prozess der Weiterentwicklung. Dies hat sich etwa in der Verlagerung des Mitteleinsatzes von massenmedialer zu personalkommunikativer Präsenz bei kontinuierlicher Zunahme der Internetkommunikation ausgewirkt (s. unten). Die zur Durchführung der 3 Kampagnen notwendigen Mittel in Höhe von jeweils rund 1 Mio. € jährlich werden aus dem Bundeshaushalt bereitgestellt. Die BZgA-Jugendkampagne „Alkohol? Kenn dein Limit.“ wird vom Verband der Privaten Krankenversicherung e. V. im Rahmen eines Sponsorings seit 2009 unterstützt. Aktuell betragen die dafür zusätzlich eingesetzten Drittmittel 4,2 Mio. € jährlich.

## Die Kampagnen zur Alkoholprävention

### „Null Alkohol – Voll Power“

Seit 2009 richtet sich die Kampagne „Null Alkohol – Voll Power“ an Kinder und Jugendliche im Alter von 12 bis 16 Jahren, um diese über die Risiken von Alkoholkonsum zu informieren und zu einer Lebensgestaltung ohne Alkohol anzuregen. In dieser Altersgruppe liegen zwar schon relevante Konsumprävalenzen vor, riskante Konsummuster haben sich aber in der Mehrheit noch nicht etabliert [3], der Erwerb sowie der Konsum von Alkohol in der Öffentlichkeit sind in diesem Alter durch das Jugendschutzgesetz zudem untersagt. Ziel der Kampagne ist es daher, den Verzicht auf Alkohol und die Verzögerung des Einstiegs in den Alkoholkonsum zu fördern [25]. Die Zielerreichung wird periodisch in den o. g. repräsentativen Studien über die Indikatoren „Lebenszeitprävalenz“ sowie „Alter bei Erstkonsum“ erhoben.

### „Alkohol? Kenn dein Limit.“ für Jugendliche

Die Kampagne „Alkohol? Kenn dein Limit.“ besteht aus 2 Teilkampagnen. Die Teilkampagne für Jugendliche und junge Erwachsene im Alter von 16 bis 20 Jahren startete ebenfalls 2009. Ab dem Alter von 16 Jahren ist der Konsum von alkoholischen Getränken in Deutschland gesetzlich erlaubt, entsprechend steigt im Jugendalter die Prävalenz des Konsums von Alkohol deutlich an: 61,9 % der 12- bis 17-Jährigen gaben 2018 an, bereits mindestens einmal in ihrem Leben Alkohol getrunken zu haben, bei den 18- bis 25-Jährigen sind es 95,1 % [3].

Der regelmäßige (mind. wöchentliche) Konsum in der Altersgruppe der 16- bis 20-Jährigen ist als riskant einzustufen, da in der Pubertät wesentliche Umbauprozesse im Gehirn stattfinden, die bis zur dritten Lebensdekade andauern [26]. Alkohol kann diese Umbauprozesse empfindlich beeinflussen und dadurch zu bleibenden Schädigungen des Gehirns führen [27, 28]. Jugendliche und junge Erwachsene sollen daher Alkohol auch in kleinen Mengen nicht regelmäßig konsumieren, sondern „weitgehend meiden“ [29]. Zudem entwickeln und verfestigen sich in der Adoleszenz gesundheitsbezogene Verhaltensweisen [30] und erlernte riskante Verhaltensweisen können zu gesundheitlichen Problemen auch im Erwachsenenalter beitragen [31]. Aus Präventionssicht ist es daher erforderlich, gezielt 16- bis 20-Jährige zu erreichen. Im Jugendalter lässt sich die Entwicklung riskanter Alkoholkonsummuster noch besser beeinflussen und ggf. korrigieren als im Erwachsenenalter, wenn sich (riskante) Alkoholkonsummuster bereits über lange Jahre gefestigt haben [32]. Die Ziele der Kampagne „Alkohol? Kenn dein Limit.“ für Jugendliche und junge Erwachsene sind daher: Rückgang des Rauschtrinkens und der Alkoholintoxikationen; Rückgang der für Erwachsene gesundheitlich riskanten Durchschnittsmengen; Rückgang des regelmäßigen Alkoholkonsums in dieser Altersgruppe. Die Zielerreichung wird periodisch in den o. g. repräsentativen Studien über Menge und Frequenz des Konsums erhoben.

### „Alkohol? Kenn dein Limit.“ für Erwachsene

Unter demselben Namen richtet sich die Teilkampagne „Alkohol? Kenn dein Limit.“ für Erwachsene seit 2000 an die Allgemeinbevölkerung ab dem Alter von 21 Jahren. Die Kampagne hat zum Ziel, den riskanten Alkoholkonsum (hohe Durchschnittsmengen, Rauschtrinken), Fälle des fetalen Alkoholsyndroms sowie abhängigen und missbräuchlichen Konsum bevölkerungsweit zu verringern. Um dies zu erreichen, soll das Problembewusstsein für riskanten Alkoholkonsum in der Allgemeinbevölkerung gesteigert werden.

Da der Schwerpunkt auf der Prävention gesundheitsschädlichen Konsumverhaltens liegt, richtet sich die Mehrebenenkampagne nicht nur an Menschen, die bereits Alkohol in gesundheitlich riskanter Weise konsumieren. Der Schwerpunkt liegt vielmehr auf jenen, die maßvoll Alkohol konsumieren – der weit überwiegende Teil der Gesamtbevölkerung –, auf Eltern aufgrund ihrer Verantwortung für den Konsum ihrer Kinder sowie auf älteren Menschen und auf suchtbelasteten Familien aufgrund deren höherer Vulnerabilität. Zur Prävention der fetalen Alkoholspektrumstörungen bzw. des fetalen Alkoholsyndroms (FAS/D) werden schwangere Frauen und ihr Umfeld darüber hinaus spezifisch angesprochen, um den Alkoholverzicht in der Schwangerschaft zu fördern. Außerdem richtet sich die Erwachsenenkampagne explizit an Fachkräfte in Beratungsstellen, Ärzte/innen sowie in der Schwangerenbetreuung Tätige und unterstützt Fachkräfte zum Thema Kinder aus suchtbelasteten Familien. Die Zielerreichung wird durch die o. g. repräsentativen Studien der BZgA sowie durch Hinzuziehen periodischer Befragungen der Allgemeinbevölkerung (Epidemiologischer Suchtsurvey [33]; Studie „Gesundheit in Deutschland aktuell“ [34]) erhoben.

### „Alkoholfrei Sport genießen“

Begleitet werden die 3 Kampagnen durch das Programm „Alkoholfrei Sport genießen“, das unter der Schirmherrschaft der Bundesdrogenbeauftragten steht. Um Multiplikatoren/innen in der Lebenswelt Freizeit/Sport spezifisch zu adressieren, hat die BZgA 2011 in Kooperation mit wichtigen Breitensportverbänden (u. a. Deutscher Fußballbund e. V., Deutscher Olympischer Sportbund e. V., Deutscher Handballbund e. V.) diese Mitmachinitiative entwickelt. Im Mittelpunkt steht die Sensibilisierung von Trainerinnen und Trainern und anderen Vereinsmitgliedern für den verantwortungsvollen Umgang mit Alkohol im Sportverein, damit sich Erwachsene beim Thema Alkohol ihrer Vorbildfunktion gegenüber Kindern und Jugendlichen bewusst sind und entsprechend handeln. Ziel des Programms ist es, die teilnehmenden Sportvereine dazu zu motivieren, Vereinsfeste, Turniere oder Ähnliches komplett alkoholfrei zu gestalten.

## Bedeutung der Internetkommunikation innerhalb der Kampagnen

Vor dem Hintergrund der zunehmenden Digitalisierung und Medialisierung der Gesellschaft bildet die Gesundheitskommunikation im Internet das zentrale Element aller BZgA-Aktivitäten zur Alkoholprävention. In den Jugendkampagnen kommt diesem Zugangsweg als Ergänzung der lebensweltlichen Präventionsansätze eine hohe Bedeutung zu, denn angesichts der verbreiteten Nutzung digitaler Medien durch Jugendliche bilden das Internet und speziell die sozialen Medien den gegenwärtig effizientesten Zugangsweg zu den Endadressaten/innen (vgl. Beitrag von Döring und Holz in diesem Themenheft).

Das Internetportal www.null-alkohol-voll-power.de dient dazu, die unter 16-Jährigen über Risiken des Alkoholkonsums zu informieren und zur Selbstreflexion anzuregen. Das Internetportal www.kenn-dein-limit.info bietet Jugendlichen qualitätsgesicherte Informationen sowie interaktive und partizipative Angebote wie Verhaltenstipps, Mitmachaktionen, Umfragen und Quizformate. Jugendliche können eine individuelle Rückmeldung zu ihrem Trinkverhalten erhalten und an dem vollautomatisierten Onlineverhaltensänderungsprogramm „Change your drinking“ teilnehmen [22]. Der Blog (blog.kenn-dein-limit.info), in dem junge Autoren/innen regelmäßig Beiträge über ihre eigenen Erfahrungen und Gedanken zum Thema Alkohol veröffentlichen, soll dem Peer-to-Peer-Ansatz folgend die Selbstreflexion bei Jugendlichen weiter anregen.

Die Erwachsenenkampagne setzt sogar primär auf die Onlinekommunikation, da die Mehrheit der Bevölkerung aller Altersgruppen das Internet nutzt und dieser Kommunikationsweg es ermöglicht, bei hoher Gesamtreichweite auch verschiedene Endadressaten/innen mit breit gefächerten Themen und Angeboten zu erreichen. Dieser Medieneinsatz erlaubt die effiziente Verwendung der finanziellen und personellen Mittel, die im Verhältnis zur Anzahl potenzieller Konsumierenden relativ gering sind.

Zentrale Inhalte des Internetportals www.kenn-dein-limit.de sind Informationen zu den Risiken des Alkoholkonsums, Anregungen zur kritischen Selbstreflexion, Unterstützung bei Alkoholverzicht/-reduktion sowie Erleichterung des Zugangs zu persönlichen Beratungsangeboten. Auf der Website stehen neben Basisinformationen weiterführende Themenrubriken mit Informationen für die verschiedenen Schwerpunkte bereit. Dort erfolgt darüber hinaus die Aktivierung der Nutzenden durch periodische Mitmachaktionen (u. a. eine Onlineaktion zum Alkoholfasten). Beratungsangebote für die erwachsene Bevölkerung werden ebenfalls niedrigschwellig und adressatenspezifisch verfügbar gemacht. Dazu unterhält die Erwachsenenkampagne eine eigene Onlineberatung zum Thema Alkoholkonsum in Ergänzung der BZgA-Telefonberatung zur Suchtprävention und macht die Datenbank der BZgA zu bundesweiten Suchtberatungsangeboten über die Website zugänglich. Um eine spezifische Onlineberatung für Schwangere zum Alkoholverzicht vorzuhalten, beteiligt sich die Kampagne am Projekt „IRIS“ (individualisierte, risikoadaptierte Intervention zur Verringerung des Alkohol- und Tabakkonsums bei Schwangeren; www.iris-plattform.de).

Jugendliche und junge Erwachsene verstehen soziale Netzwerke zunehmend nicht nur als Kommunikationsmittel, sondern als Informationsquelle. Jugendliche nutzen die Plattformen Youtube und Facebook bereits deutlich häufiger als Webangebote klassischer Zeitungen oder Zeitschriften, um sich zu informieren [35]. Soziale Netzwerke ermöglichen es in besonderem Maße, in einen Dialog zu treten. Ziel ist nicht nur die Wissensvermittlung, sondern auch die Förderung kritischer Kommunikation unter den Konsumierenden sowie die Anregung zum Hinterfragen eigener Konsummotive. In den sozialen Medien wird Alkoholkonsum jedoch oft ironisch und affirmativ dargestellt und kommentiert [36, 37]. Strategisch sind die Social-Media-Kanäle der beiden Kampagnen „Alkohol? Kenn dein Limit.“ auf Facebook (www.facebook.com/alkoholkenndeinlimit, www.facebook.com/kenndeinlimiterwachsene), Twitter (twitter.com/Alkohol_Limit), YouTube (www.youtube.com/alkoholkenndeinlimit) und Instagram (www.instagram.com/alkohol_kenndeinlimit) daher darauf ausgerichtet, in den sozialen Medien eine Gegenöffentlichkeit zu entwickeln und damit, vor allem im Jugendalter, kritische Einstellungen gegenüber Alkohol zu stärken. Hinsichtlich der erwachsenen Adressaten/innen streben die Kampagnen primär einen Beitrag zur Normkorrektur an (kritische Haltung, realistische Einschätzung der Risiken, niedrigen Konsum und Abstinenz stärken). Hierzu veröffentlichen die Kampagnen mehrmals wöchentlich Beiträge, beispielsweise kurze Videoclips, die sich an aktuellen Themen und Nutzerverhalten orientieren.

Spezifisch an Fachkräfte richten sich die Onlineangebote www.alkoholfrei-sport-geniessen.de für Sportvereine und die Serviceplattform www.vortiv.de („Vor Ort aktiv“) für kommunale Fach- und Steuerungskräfte, die alle Angebote der BZgA zur kommunalen Alkoholprävention bündelt.

Alle Maßnahmen der digitalen Gesundheitskommunikation werden durch Suchmaschinen- und Social-Media-Marketing gestützt, um die einfache Verfügbarkeit für die Endadressaten/innen sowie eine hohe Reichweite sicherzustellen.

## Bedeutung der Personalkommunikation innerhalb der Kampagnen

Personalkommunikation hat die Funktion, im persönlichen Kontakt individuell auf Interessen und Wissenslücken einzugehen sowie Vorbildlernen zu ermöglichen, um damit gewünschtes Gesundheitsverhalten anzuregen oder zu festigen. Die ressourcenintensiven personalkommunikativen Maßnahmen in den Lebenswelten Schule, Kommune, Freizeit und Sport werden ausschließlich im Rahmen der beiden BZgA-Jugendkampagnen eingesetzt, um bereits bei Kindern und Jugendlichen gesundes Verhalten zu fördern. Die Kampagne „Null Alkohol – Voll Power“ erreicht Schüler/innen mit der „Voll Power-Schultour“, einer bundesweit durchgeführten personalkommunikativen Maßnahme mit erlebnispädagogischen Elementen im Setting Schule. Die Schultour erreicht Schulen, die im Vorfeld bereits eine alkoholpräventive Maßnahme durchgeführt haben, und stärkt so präventive Strukturen. Sie wird ergänzt durch Arbeitshilfen für Multiplikatoren/innen (einschl. einer „Aktionsbox“). Die Kampagne beteiligt sich außerdem an der bundesweiten Tour des Mitmachparcours „KlarSicht“ zur schulischen Alkohol- und Tabakprävention sowie am Angebot „JugendFilmTage“ [38].

Durch die Kampagne „Alkohol? Kenn dein Limit.“ für Jugendliche werden seit 2011 kommunale Fachkräfte bei der qualitätsgesicherten und ressortübergreifenden Netzwerkarbeit in den Kommunen unterstützt, deren strukturelle Weiterentwicklungen gefördert und Fachkräfte entsprechend fortgebildet. Die schulischen Angebote „JugendFilmTage“ und „KlarSicht“-Mitmachparcours – als Koffervariante – wurden im Rahmen der Jugendkampagne weiterentwickelt und stehen nun zur eigenständigen Durchführung in Kommunen zur Verfügung. In der Lebenswelt Schule führt die Kampagne den bundesweiten Klassenwettbewerb „Klar bleiben – Feiern ohne Alkoholrausch“ durch, beteiligt sich am o. g. Mitmachparcours „KlarSicht“ und stellt für Lehrkräfte Handreichungen und Aktionsmaterialien bereit.

Im Freizeitbereich sind Peer-Edukatoren/innen der BZgA im Alter von 18 bis 24 Jahren seit 2009 bundesweit im Rahmen der Jugendkampagne „Alkohol? Kenn dein Limit.“ im Einsatz, um mit Jugendlichen persönlich ins Gespräch zu kommen. Die Kampagnen-Peers werden in ganz Deutschland pro Jahr ca. 200-mal dort eingesetzt, wo Jugendliche in ihrer Freizeit unterwegs sind. Auch auf großen Sport- und Freizeitveranstaltungen sind die beiden Jugendkampagnen u. a. mit einem Eventareal vertreten. Alkoholfreie Aktivitäten von Sportvereinen unterstützt die Mitmachinitiative „Alkoholfrei Sport genießen“ bundesweit in Kooperation mit Breitensportverbänden und die BZgA unterstützt die Initiative „Doppelpass 2020“ des Deutschen Fußball-Bundes e. V. zur Stärkung der Zusammenarbeit von Schulen und Vereinen mit Materialien aus den Jugendkampagnen.

## Bedeutung von Printmedien und Massenkommunikation innerhalb der Kampagnen

Begleitet werden die bisher genannten Angebote der Kampagnen durch Massenkommunikation in Form von Plakaten, Spots, Anzeigen sowie durch Printmedien. Massenmedien, neben bundesweiten Plakatschaltungen u. a. Kino- und TV-Spots sowie Ambientmedien (verschiedenartige Werbeträger im Außenbereich), wurden vor allem zu Beginn der Jugendkampagne „Alkohol? Kenn dein Limit.“ ab 2009 strategisch eingesetzt, um das Thema in der Öffentlichkeit zu platzieren, Problembewusstsein zu schaffen und um zu gesellschaftlichen Debatten, wie dem Gesundheitszieleprozess, beizutragen. Der Einsatz dieser Medien dient auch weiterhin dazu, die hohe Bekanntheit bei den Endadressaten/innen (2018: 84 % der 12- bis 25-Jährigen [15]) sowie bei Multiplikatoren/innen und Fachöffentlichkeit zu sichern und auszubauen sowie die hohe Akzeptanz beizubehalten. Denn es ist plausibel, dass Kampagnenmaßnahmen in den Lebenswelten besser wahrgenommen und Angebote besser angenommen werden, wenn der Absender, d. h. die BZgA und die Kampagnen „Alkohol? Kenn dein Limit.“, bekannt und bei den Endadressaten/innen, aber auch bei Multiplikatoren/innen als fachlich kompetent akzeptiert ist.

Printmedien dienen der Förderung von Gesundheitskompetenzen bei den Endadressaten/innen, indem sie eine intensivere Beschäftigung und Auseinandersetzung mit dem Thema Alkohol ermöglichen. Sie liegen entweder als kurze Faltblätter mit Basisinformationen zum jeweiligen Thema vor mit der Funktion, auf die Onlinethemenrubrik sowie auf weiterführende Printmaterialien hinzuweisen. Gleichzeitig existieren ausführliche thematische Broschüren, die eine eingehende Auseinandersetzung ermöglichen. Diese eignen sich besonders für die Ausgabe durch Fachkräfte (in Präventionsberatungsstellen, Schulen usw.). Spezifisch für das Fachpublikum werden Printmedien entwickelt, um (Kurz‑)Interventionen zur Reduktion des Alkoholkonsums durch Fachkräfte zu unterstützen. Zurzeit liegen Manuale für Lehrkräfte, für Sporttrainer/innen, für Ärzte/innen zur Ansprache riskanten Alkoholkonsums und zur Beratung Schwangerer zum Alkoholverzicht vor, deren Inanspruchnahme auch durch Anzeigenschaltungen unterstützt wird.

Die Printmaterialien sind über das Bestellsystem der BZgA kostenfrei für Einzelpersonen und Institutionen erhältlich. Im Jahr 2020 wurden insgesamt knapp 500.000 Exemplare zum Thema Alkohol ausgegeben. Ergänzt wird dieses Angebot, indem die BZgA weitere Publikationen der Deutschen Hauptstelle für Suchtfragen zum Thema Alkohol gezielt fördert.

## Fazit

In den Maßnahmen zur Alkoholprävention der BZgA wirken Interventionsformen auf verschiedenen Ebenen zusammen, die adressatengerecht ausdifferenziert sind, sich ergänzen und miteinander Synergien erzielen. Die Gesamtheit der dargestellten Angebote kann als komplexe Intervention verstanden werden. Kausale Wirknachweise sind hierfür schwer zu erbringen, jedoch sind die einzelnen Interventionsformen qualitätsgesichert, wenn möglich wurde die Wirksamkeit von Einzelmodulen mit randomisiert kontrollierten Studien überprüft (s. oben und z. B. den Beitrag von Hanewinkel et al. in diesem Themenheft). Alle Einzelmaßnahmen werden fortlaufend weiterentwickelt, etwa indem als Reaktion auf Veränderungen des jugendlichen Kommunikationsverhaltens zunächst ein Facebook-, dann ein Instagram-Kanal eingerichtet wurde. Das Gesamtpaket wird laufend in Kooperation mit Partnern der Suchtprävention in Bund, Ländern und Kommunen auf Ergänzungs- und Optimierungspotenzial geprüft. Die aktuellen Entwicklungen des Alkoholkonsums in der Altersgruppe 12 bis 25 Jahre werden durch das periodische Monitoring der BZgA (Drogenaffinitätsstudie, s. oben) untersucht.

Bereits jetzt wirken verhaltenspräventive und strukturelle Interventionen in bestimmten Settings, wie etwa Schule und Kommune im Sinne des Lebensweltansatzes, zusammen (vgl. Beitrag von Praßer et al. in diesem Themenheft). Dennoch besteht in Fachkreisen Einigkeit, dass eine Kombination mit evidenzbasierten strukturellen Interventionen, wie einer restriktiveren Verfügbarkeits- und Preispolitik, zu einer höheren Wirksamkeit der in der Verhaltensprävention eingesetzten Mittel führen würde. Die Erfolge in der Tabakprävention haben die Potenziale eines solchen „Policy-Mix“ (kombinierter Einsatz verschiedener Maßnahmen) für die Situation in Deutschland gezeigt [38, 39]. Aus Sicht der BZgA könnten verhaltenspräventive Kampagnen einen wichtigen Beitrag dazu leisten, zukünftige verhältnispräventive Maßnahmen kommunikativ einzubetten und der Bevölkerung zu vermitteln, sofern der politische Wille dazu vorhanden ist. Und nicht zuletzt ist die bundesweite Alkoholprävention wichtig, weil sie die Funktion des Agendasettings erfüllt, also neben dem individuellen Verhalten der Endadressaten/innen sowohl deren Bewusstsein für die Bedeutung des Themas als auch politische Entscheidungen beeinflussen und die Präventionsarbeit vor Ort unterstützen kann.

Alkoholprävention muss langfristig angelegt sein, denn schnelle Auswirkungen sind, unter anderem bedingt durch einen sich nur sehr langsam wandelnden gesellschaftlichen Umgang mit dem Alkoholkonsum, nicht zu erwarten. Damit bleibt die kontinuierliche, bevölkerungsweite Alkoholprävention eine zentrale Aufgabe innerhalb der BZgA-Suchtprävention.
